# Diabetes mellitus and associated factors among HIV-positive patients at primary health care facilities in Harare, Zimbabwe: a descriptive cross-sectional study

**DOI:** 10.1186/s12875-024-02261-3

**Published:** 2024-01-15

**Authors:** Rumbidzai Chireshe, Tawanda Manyangadze, Keshena Naidoo

**Affiliations:** 1 Discipline of Public Health Medicine, College of Health Sciences, University of KwaZulu-Natal, Howard Campus, Mazisi Kunene Road, Glenwood, Durban, 4041 South Africa; 2 2Department of Geosciences, School of Geosciences, Disasters, and Development, Faculty of Sciences and Engineering, Bindura University of Science Education, Bindura, Zimbabwe; 3 Department of Family Medicine, School of Nursing and Public Health, University of KwaZulu-Natal, Durban, South Africa

**Keywords:** Type 2 Diabetes Mellitus, DM prevalence, HIV, Non-communicable diseases, PLWH, Primary healthcare, Sub-Saharan Africa, Zimbabwe

## Abstract

**Background:**

Highly active antiretroviral therapy (HAART) has improved the life expectancy of people living with HIV (PLWH) and has increased the risk of chronic non-communicable diseases. Comorbid HIV and diabetes mellitus (DM) significantly increase cardiovascular disease and mortality risk. This study aimed to determine the prevalence of type 2 diabetes mellitus among HIV-positive patients receiving HAART in Zimbabwe and its associated risk factors.

**Methods:**

This cross-sectional study was conducted at eight primary healthcare facilities in Harare, Zimbabwe, between January 2022 and March 2023. Non-probability convenience sampling was used to recruit adult HIV-positive patients undergoing HAART attending the facilities. Data were captured on clinical history and socio-demographic and behavioral characteristics, and analyzed using descriptive statistics to determine DM prevalence rates. Additionally, bivariate and multivariate logistic regression models were employed to examine factors associated with HIV and DM comorbidities.

**Results:**

A total of 450 participants were included in this study, of which 57.6% (*n* = 259) were female. The majority were married (73.8%) and older than 35 years (80.2%). Most participants had completed high school (87.6%) and 68.9% were employed either formally or self-employed. The prevalence of diabetes mellitus (DM) was 14.9%. HIV/DM comorbidity was more prevalent in patients who were female, self-employed, and smoked (*p* < 0.05). Multivariate logistic regression analysis revealed that the factors associated with DM-HIV comorbidity were gender, age, education, marital status, employment status, smoking, physical activities, duration of HAART, and diet. Age, level of education, marital status, and occupation were not associated with HIV-DM comorbidity. Obesity (body mass index > 30 kg/m2), smoking, and alcohol consumption were associated with an increased risk of DM. Regular physical activity is associated with a reduced risk of DM.

**Conclusion:**

A substantial burden of DM was found in PLWH. The intersectoral integration approach is advocated, and active screening for DM is recommended. Gender-specific interventions are necessary to target diseases and health behaviors that differ between men and women. These interventions should be customized to the specific diseases and behaviors of each group.

**Supplementary Information:**

The online version contains supplementary material available at 10.1186/s12875-024-02261-3.

## Introduction

The burden of disease from HIV remains high, with an estimated 37.7 million individuals globally living with HIV, as reported in 2020, and over 1.5 million people contracting HIV in 2020 alone [1]. Sub-Saharan Africa (SSA) has the greatest burden of HIV. Despite significant improvements in HIV-related morbidity and mortality following the introduction of highly active antiretroviral therapy (HAART), people living with HIV (PLWH) are vulnerable to other co-morbidities such as diabetes mellitus [2–6]. Diabetes Mellitus (DM), an illness resulting from either insulin resistance or deficiency that leads to abnormally elevated blood glucose levels, is one of the most prevalent Non-Communicable Diseases (NCD) worldwide and in Africa [7]. According to previous reports, the prevalence of DM is estimated to range from 2.6% in Togo to 22.5% in Niger, depending on the region. [8] DM was responsible for a 7.0% mortality rate attributed to NCDs in SSA between 1990 and 2015 [9, 10].

In response, the World Health Organization (WHO) has recommended that primary healthcare (PHC) systems provide screening and treatment for NCDs and integration of HIV and primary health systems (PHC) [11]. However, there is limited evidence on the prevalence of diabetes among PLWH at the primary care level in SSA, and the response of health systems to address comorbidities among PLWH. Although world leaders pledged the United Nations General Assembly to address NCDs, the issue of HIV-NCD multimorbidity has not been specifically addressed. According to the World Health Organization's (WHO) Global Action Plan for the Control of NCDs 2013–2020, nations should improve their health systems and combat NCDs by implementing universal health coverage (UHC) and person-centered PHC [12]. Despite attempts by some African countries, such as Ghana and Nigeria, to attain Universal Health Coverage through universal health insurance programs, most populations in Africa access healthcare at government-funded health facilities or pay out-of-pocket. People living with multiple morbidities are further compromised by multiple visits and services from the health sector.

However, there has been some progress towards attaining the Sustainable Development Goal target 3.4, which states that "by 2030, premature mortality from NCDs shall be reduced by one-third through prevention and treatment and be promoted to promote mental health and well-being’ [13, 14]. People living with HIV have benefitted from recommendations such as the WHO Package of Essential Noncommunicable Disease Interventions (WHO PEN), which have been combined to provide information on the methodologies for the management of co-infections and comorbidities, focusing on the screening, prophylaxis, treatment, and timing of ART for these conditions [15]. The WHO STEPwise chronic disease risk factor surveillance program (STEPS) attempts to quantify the burden of diabetes in sub-Saharan Africa (SSA). A surveillance program is a straightforward, standardized strategy for gathering, examining, and sharing information on important NCD risk factors in countries. It is a questionnaire-based evaluation that considers both the biochemical and physical parameters. The national authorities of the country implement a coordinated strategy [16].

Several risk factors for DM in PLWH have been identified in previous studies. These include a family history of diabetes, weight gain, lipodystrophy, advanced age, and hepatitis C infection among PLWH receiving protein inhibitor therapy [17]. A noteworthy correlation between diabetes and the duration of antiretroviral therapy was also identified, particularly the use of protease inhibitor drugs [18, 19]. The extended time spent living with HIV, persistent low levels of inflammation, oxidative stress, and mitochondrial damage caused by HIV medication put PLWH at a slightly greater risk of developing diabetes [20]. It has been observed that PLWH taking HAART have a four-fold increased risk of developing diabetes mellitus compared to those without HIV [6, 21, 22]. The underlying mechanisms are believed to be either a direct result of drug side effects or an indirect consequence of immune system reactivation and the subsequent acquisition of health [19]. These mechanisms permit and enhance the impact of conventional risk factors that are independent of HIV infection, such as advanced age, obesity, smoking, sedentary lifestyle, family history, and genetic predisposition [23–27].

The direct effects of HIV infection on several organ systems, the toxicity of ART, polypharmacy, social isolation, stigma, and other poorly identified risk factors are only a few of the many factors that likely affect the health of HIV-infected adults. The added burden of another chronic condition, such as DM, adversely affects quality of life [28]. In HIV patients, diabetes has been linked to an increased risk of hospitalization, adverse cardiovascular and renal outcomes, and progression to end-stage renal disease, leading to reduced life expectancy and higher treatment costs for this population [5]. Although there is a paucity of reliable information regarding NCD in some resource-constrained clinics and suboptimal NCD care in clinics, the results from our study will highlight these gaps and help guide policymakers and stakeholders in the synchronization of actions to help improve the quality of life for patients with multimorbidity.

This study reports the prevalence of diabetes mellitus (DM) among HIV-infected patients receiving primary health care services at primary care clinics in Harare, Zimbabwe, and associated factors. The findings are intended to identify potential risk factors for DM/HIV comorbidity and potential interventions to improve the integration of care.

## Methodology

### Study setting

The study setting was primary healthcare clinics (PHC) in Harare, the capital city of Zimbabwe. Clinics provide primary healthcare services for a population of approximately 2.5 million people in urban and rural communities [29]. Eight (8) primary health clinics were selected to represent urban and rural communities. Six of the facilities were located in urban areas, and two were in rural areas.

The majority of PHC facilities in the region are funded by the government or non-governmental organizations (NGOs) and provide free access to all people requiring primary care services. These PHC clinics provide a wide range of services such as HIV screening, diabetes and hypertension management, acute and chronic condition management, and health promotion. During each visit, routine blood pressure, weight, temperature, and urine composition measurements were recorded. According to the national guidelines, patients with classic symptoms can be diagnosed with diabetes mellitus (DM) by measuring their plasma glucose levels. Tests, such as the fasting plasma glucose (FPG) test, measure blood glucose after a minimum of eight hours without food or liquids other than water. To screen for diabetes, two blood tests were used: the random plasma glucose test, which measures the blood glucose level when you have not fasted for at least eight hours, and the HbA1C test, which gives an average of your blood glucose levels over the previous two–three months. An abnormal glycosylated hemoglobin test or two abnormal fasting glucose readings were used to diagnose diabetes.

As is the case with most diagnostic tests, if a test result indicates diabetes and the diagnosis is unclear because of laboratory error, it should be repeated. An example would be a patient who exhibited classic hyperglycemia or hyperglycemic crisis symptoms. Because there is a higher chance of concurrence in this instance, it is preferable to repeat the test for confirmation.

Professional nurses and medical officers provided the clinical services. Most continuing clinical care is provided by registered general nurses who have received additional training in HIV management. With the assistance of the hospital's radiology and laboratory departments for diagnostic support, doctors offered supplemental care on a consultation basis. Both paper-based and electronic files are used to store patient data, which are typically linked by means of distinct patient identification numbers. Every clinic visit included the recording of patient information on the stage of HIV disease, incident AIDS-related and non-AIDS illnesses, prescribed medications, and laboratory test results.

### Study design

We conducted a cross-sectional descriptive study at primary healthcare facilities in Harare, Zimbabwe. A mixed-method approach was used to collect quantitative data through data extraction from patient records and qualitative data through semi-structured patient interviews.

Data extracted from patient records included participant demographics, weight, height, past medical history, antiretroviral medication history, and current illnesses, including communicable and non-communicable diseases.

### Study population

The population of interest was adult HIV-positive patients receiving primary care services in Harare, Zimbabwe.

#### Inclusion criteria

- HIV-positive adults aged 18 years and older
- Registered as patient at the study site.
- Receiving primary health services

#### Exclusion criteria

- HIV-negative
- Unable to provide consent.
- Not registered as a patient at a study site

### Sample size and sampling procedure

Purposive sampling was used to recruit the participants who met the inclusion criteria.

The minimum sample size was determined using a single population proportion formula to calculate the prevalence of diabetes in the HIV patient population.

This formula is represented as

$$=\frac{{(Z1-a)}^{2}\times p(1-p)}{{d}^{2}}$$

where $z=1.96$ (critical value at a certain confidence interval in a normal distribution scenario), $p=50\%$ (the expected proportion of the prevalence of type 2 diabetes mellitus (T2DM)/HIV), and $Margin of error (d=0.05)$ (the desired level of absolute precision, taken as 0.05). The values of the parameters in the above formula were determined as follows:

$$n=\frac{{1.96}^{2}\times 0.5\times 0.5}{0{.05}^{2}} =384$$

Assuming that 10% will decline to participate in the study, the minimum sample size is given as:

$$n=384+ 10\mathrm{\% }\left(38.4\right)=422.4 \approx 423$$

Therefore, a minimum sample size of 423 was used in this study.

Eligible participants were identified from the clinic register and recruited from each of the eight clinics until the sample size was met. The data were collected between January 2022 and March 2023. Clinics 1 to 6 provided services to an urban community, while clinics 7 and 8 were situated in rural/per-urban communities. Clinic 1 had 68 participants, Clinic 2 had 45 participants, Clinic 3 had 53 participants, Clinic 4 had 62 participants, Clinic 5 had 45 participants, Clinic 6 had 70 participants, and rural/peri-urban clinics included Clinic 7 and Clinic 8 with 52 and 55 participants, respectively. A total of 450 participants were recruited for the study.

### Data collection methods

Both secondary and primary data collection methods were used in this study, as explained in the following sections. The participants were allocated a study code and no names were recorded.

#### Reviewing clinical records

Body Mass Index (BMI) was calculated from the data extracted from the clinical records. BMI was recorded and classified as underweight (< 18.5 kg/m^2^), normal (18.5 kg/m^2^ – 24.9 kg/m^2^), overweight (25.0 kg/m^2^ – 29.9 kg/m^2^), or obese (≥ 30 kg /m^2^). For the analysis, two categories of BMI were considered: obese (≥ 30 kg /m^2^) and non-obese (< 30 kg /m^2^).

#### Structured questionnaire

The pilot-tested structured questionnaire served as the primary means by which the data were collected. It was designed to collect information on whether a patient had DM and on the possible risk factors associated with DM in PLWH. The WHO STEPS framework was utilized as a standardized and versatile instrument for identifying non-communicable disease risk factors, including the demographic, behavioral, and clinical features of participants. The sociodemographic variables of the participants included age, gender, marital status, level of education, and employment. The captured behavioral factors included smoking, alcohol consumption, dietary intake, and physical activity. Clinical features included duration of HAART, BP, blood glucose, height, and weight measurements.

### Data analysis

Data analysis was performed using Stata version 17. Descriptive statistics in the form of frequencies and percentages were used to provide a summary of DM/HIV comorbidity and independent variables. The characteristics of HIV-positive patients with DM were described and compared with those of HIV patients without DM comorbidity. Chi-square tests were used to evaluate the existence of an association between risk factors and the DM/HIV comorbidity status. This study constructed bivariate and multiple logistic regression models to ascertain the risk factors associated with DM/HIV comorbidity. A bivariate logistic regression model was used to ascertain whether a given predictor variable affected DM/HIV comorbidity, while multiple regression models controlled for other risk factors. The decision on coefficients was made using *p*-values at the 5% significance level (*P* < 0.05).

Independent variables were categorised into three groups: socio-economic, behavioural, and biological factors based on the literature review [17, 23–25, 30–34]. Socioeconomic factors included age (coded as 1 for ≥ 30 and 0 for < 35), marital status (coded as 1 = married, 2 = single, and 3 = widow/divorced), education (coded as 1 = primary or less, 2 = secondary education, and 3 = tertiary education), employment status (coded as 1 = unemployed, 2 = self-employed, and 3 = formally employed), and gender (coded as 1 = male and 0 = female). As for the risk factors, BMI was classified as 1 = obese (≥ 30 kg /m^2^) and not obese (< 30 kg /m^2^). Smoking was classified as 'yes' for smokers (coded as 1) and 'no' for nonsmokers (coded as 0). Alcohol consumption was divided into two categories: 'yes' for patients who consumed alcohol (coded as 1) and 'no' for those who did not consume alcohol. Fruit and vegetable consumption was entered into the analysis as a dummy variable, taking a value of 1 for patients with fruit and vegetable servings at least once a day and zero for patients who rarely take fruit and vegetable servings. Fried/fast food was included as binary, taking a value of 1 if the patient ate fried/fast food at least once per day, and 0 if the patient rarely consumed fried/fast food. Physical activity had two categories: 'yes' (coded as 1 for patients involved in sports and exercises) and 'no' (coded as 0 for patients not involved in sports and exercises). Following previous research, such as Duguma et al. [17], the research included hypertension as a predictor, which had two categories: 'yes' (coded as 1 for patients with hypertension) and 'no' (coded as 0 for patients with no hypertension).

## Results

### The prevalence of T2DM among HIV-positive patients

A total of 450 participants were included in the study, of which 76.2% resided in urban communities and 23.8% resided in rural communities. The prevalence of Diabetes Mellitus (DM) among HIV-positive patients in PLWH receiving primary care services at local clinics in Harare was 14.9% (*n* = 67). Urban healthcare clinics had a prevalence of 14%, whereas rural healthcare clinics had a prevalence of 0.9%.

The prevalence of diabetes among PLWH was significantly higher in participants from urban areas (14%) than in those from rural clinics (0.9%). There was a gender disparity, with 57.6% of the participants identifying as female. Notably, most participants were over 35 years old (80.2%), while the rest were aged between 18–35. The proportion of married respondents was the highest (73.8%). The level of employment was high with approximately half of the participants being self-employed (49.8%). In terms of BMI, 17.8% of the participants were categorized as obese. Table 1 shows that most participants did not consume alcohol (53.1%), 78.9% did not smoke tobacco cigarettes, and 73.6% were involved in sports, fitness, or recreational activities at least once per day. Furthermore, 49.8% had attended secondary school, 37.8% had attended higher education, 8.7% had attended primary education, and 3.8% had no education. The longer a patient was on HAART, the higher the chances of developing diabetes, and the likelihood of developing DM was higher in patients with comorbid hypertension.

**Table 1:** Socio-demographic and behavioural characteristics of participants with and without T2DM co-morbidity

**Variables**	**Total (*N* = 450)**	**DM/HIV status**		${{\varvec{\chi}}}^{2}$	***p-*value**
		**HIV only N (%)**	**DM/HIV N (%)**		
**Residence**					
Rural	107 (23.8)	103 (26.9)	4 (6.0)	13.77	< 0.05*
Urban	343 (76.2)	280 (73.1)	63 (94.0)		
**Gender**					
Women	259 (57.6)	209 (54.6)	50 (74.6)	9.4	< 0.05*
Men	191 (42.4)	174 (45.4)	17 (25.4)		
**Age**					
≤ 35	89 (19.8)	71 (18.5)	18 (26.9)	2.5	0.05*
> 35	361 (80.2)	312 (81.5)	49 (73. 1)		
**Marital status**					
Married	332 (73.8)	302 (78.9)	30 (44.8)	34.2	0.05*
Unmarried	118 (26.2)	81 (21.1)	37 (55.2)		
**Education**					
No education	17 (3.8)	17 (4.4)	0 (0.0)	15.3	0.05*
Primary education	39 (8.7)	39 (10.2)	0 (0.0)		
Secondary	224 (49.8)	193 (50.4)	31 (46.3)		
Higher education	170 (37.8)	134 (35.0)	36 (53.7)		
**Occupation**					
Unemployed	140 (31.1)	130 (33.9)	10 (14.9)	24.5	0.05*
Self-employed	224 (49.8)	172 (44.9)	52 (77.6)		
Formally employed	86 (19.1)	81 (21.1)	5 (7.5)		
**BMI**					
Obese	80 (17.8)	75 (19.6)	5 (7.5)	5.7	0.05*
Not obese	370 (82.2)	308 (80.4)	62 (92.5)		
**Alcohol**					
No	239 (53.1)	193 (50.4)	46 (68.7)	7.6	0.05*
yes	211 (46.9)	190 (49.6)	21 (31.3)		
**Smoking**					
No	355 (78.9)	293 (76.5)	62 (92.5)	8.8	0.05*
Yes	95 (21.1)	90 (23.5)	5 (7.5)		
**Fast foods/fried foods**					
Rarely eat	353 (78.4)	300 (78.3)	53 (79.1)	0.02	0.887
At least five times a day	97 (21.6)	83 (21.7)	14 (20.9)		
**Fruits and vegetables**					
Rarely eat	77 (17.1)	67 (17.5)	10 (14.9)	0.3	0.607
At least once a day	373 (82.9)	316 (82.5)	57 (85.1)		
**Exercising**					
No	331 (73.6)	271 (70.8)	60 (89.6)	10.4	0.05*
Yes	119 (26.4)	112 (29.2)	7 (10.4)		

^*^Statistically significant at *p* ≤ 0.05

In this study, a significantly greater proportion of women (74.6%) had DM/HIV comorbidity than males. Patients with higher education (53.7%) had DM/HIV comorbidity than those who had no education or primary or secondary level education. A significantly greater proportion of patients aged > 35 years (73.1%) had DM/HIV comorbidity than patients aged < 35 years. Those who were unmarried (55.2%) had a larger proportion of patients with DM/HIV comorbidity than those who were married. There was a significantly greater proportion of self-employed individuals (77.6%) than those who were unemployed or formally employed. A smaller percentage of patients with DM/HIV comorbidity were obese (7.5%) than their counterparts because the risk of DM increases linearly with BMI. There was a significant association between DM comorbidity and gender, education, alcohol consumption, exercise, BMI, occupation, and smoking (*P* < 0.05). However, the results indicated that there was no association between having DM/HIV and patients’ servings of fruits and vegetables (*P* > 0.05) among all the risk factors.

The findings in Table 1 indicate that participants from urban communities were significantly more likely to have DM/HIV comorbidity than those from rural communities. Further analysis was conducted on the differences between urban and rural dwelling participants with DM/HIV comorbidities (Table 2).

**Table 2:** Simple and multiple logistic regression models for DM/HIV co-morbidity

Independent variables	COR	*p*-value	AOR	*p*-value
**Residence**				
Rural	1		1	
Urban	5.8 [2.1, 16.3]	0.00	5.5 [1.9, 15.7]	0.05*
**Gender**				
Female	1		1	
Male	0.4 [0.2, 0.7]	0.05*	0.4 [0.2, 0.9]	0.05*
**Age**				
≤ 35	1		1	
> 35	0.6 [0.3, 1.1]	0.12	0.1 [0.1, 0.3]	0.05*
**Education**				
Secondary and lower	1		1	
Higher education	2.2 [1.3, 3.6]	0.05*	8.6 [3.2, 23.0]	0.05*
**Marital status**				
Married	1		1	
Unmarried	3.2 [1.6, 6.3]	0.05*	5 [3.2, 7.8]	0.05*
**Religion**				
Non-Christianity	1		1	
Christianity	2.3 [0.9, 5.9]	0.09	1.1 [0.3, 4.0]	0.96
**Employment status**				
Unemployed	1		1	
Self-employed	3.9 [1.9, 8.0]	0.05*	6.4 [2.9, 13.9]	0.05*
Formally employed	0.8 [0.3, 2.4]	0.50	0.6 [0.2, 2.2]	0.05*
**Smoking**				
No	1		1	
Yes	0.3 [0.1, 0.7]	0.01*	0.02 [0.006, 0.1]	0.05*
**Alcohol**				
No	1		1	
Yes	0.5 [0.3, 0.8]	0.01*	2.0 [0.9, 4.8]	0.05*
**Fruits & Vegetables**				
No	1		1	
Yes	1.2 [0.6, 2.5]	0.61	0.5 [0.2, 1.1]	0.05*
**Fried & Fast food**				
No	1		1	
Yes	1.0 [0.5, 1.8]	0.89	0.6 [0.3, 1.5]	0.30
**Exercise**				
No	1		1	
Yes	0.3 [0.1, 0.6]	0.05*	0.3 [0.1, 0.9]	0.05*
**BMI**				
Non-obese	1		1	
Obese	0.3 [0.1, 0.9]	0.02*	0.7 [0.2, 2.7]	0.62
**Duration on HAART**	1.1 [1.0, 1.2]	0.05*	1.1 [1.0, 1.3]	0.05*
**Hypertensive**				
Non- Hypertensive	1		1	
Hypertensive	3.2 [1.9, 5.5]	0.05*	8.4 [3.5, 20.6]	0.05*
Constant	-	-	0.01 [0.001, 0.009]	0.00

*Ref* reference category, *CI* Confidence Interval, *COR* Crude Odds Ratios, *AOR* Adjusted Odd Ratios

^*^statistically significant at *p* ≤ 0.05

### Logistic regression results of risk factors of T2DM/HIV

Table 2 displays the outcomes derived from the binary logistic regression model for risk factors associated with DM among HIV-positive individuals.

### Demographic factors

In comparison to their female counterparts, male participants were less likely to develop Diabetes Mellitus (Adjusted Odds Ratio (AOR): 0.5, *p* < 0.05, 95% Confidence Interval [CI]: 0.2–1.0). HIV-positive patients older than 35 had a lower likelihood of developing DM compared to their counterparts (AOR: 0.2, *p* < 0.05, 95% CI: 0.1–0.3).

### Socio-economic factors

HIV-positive individuals who were self-employed displayed a likelihood of contracting Diabetes Mellitus when juxtaposed with their jobless counterparts (AOR: 6.4, *p* < 0.05, 95% CI: 2.9–13.9). Compared to HIV-positive patients with secondary education and less, patients with higher education were more likely to develop DM (AOR: 8.6, *p* < 0.05, 95% CI: 3.2–23.0).

### Behavioural risk factors

Table 2 presents findings indicating that individuals who are HIV-positive and consume tobacco products are at a decreased risk of developing Diabetes Mellitus (DM) in comparison to non-tobacco users (AOR: 0.02, *p* < 0.05, 95% CI: 0.006–0.1). Conversely, individuals who are HIV-positive and abstain from alcohol consumption are at a heightened risk of developing DM in comparison to those who do consume alcohol (AOR: 2.0, *p* < 0.1, 95% CI: 0.9–4.8). Additionally, physical activity in the form of sports and fitness activities is linked with a reduced risk of developing DM/HIV comorbidity (AOR: 0.3, *p* < 0.05, 95% CI: 0.1–0.9). In terms of dietary habits, HIV-positive individuals who consumed fruits and vegetables at least five times a day exhibited a lower risk of developing DM in comparison to those who rarely consume fruits and vegetables (AOR: 0.5, *p* < 0.05, 95% CI: 0.2–1.1).

### Duration on HAART

Individuals who have been on HAART for more than 2 years are most likely to develop DM/HIV comorbidity (AOR: 1.1, *p* < 0.05, 95% CI: 1.0–1.3).

### Hypertension

HIV-positive individuals who were hypertensive displayed a likelihood of having Diabetes Mellitus when juxtaposed with their non-hypertensive counterparts (AOR: 8.4, *p* < 0.05, 95% CI: 3.5–20.6).

## Discussion

Historically, PHC facilities in Zimbabwe have focused on the management of single disease entities, with a limited inclusion of screening for NCDs among PLWH. The adoption of integrated chronic care models has improved the detection and management of NCDs in PLWH. However, an improved understanding of key risk factors could improve disease prevention strategies and targeted community surveillance.

Our study demonstrated a relatively high prevalence of DM/HIV comorbidity in Harare in Zimbabwe compared to other regions in Africa. This estimated prevalence rate is notably greater than those reported in comparable studies carried out in the SSA region, which typically showed rates between 2 and 14% [35]. In Ethiopia, studies have shown that the prevalence of DM in individuals living with HIV is 8% [25] and 11.4% [17], respectively, which could be because obesity is not a health problem in Ethiopia [36]. Our findings further surpassed the prevalence rates of other studies conducted in Zimbabwe (2.83%) by Magodoro et al. [37], 6.9% established by Cheza et al. [32], and 8.4% by Gonah et al. [33] could be because our study was conducted in urban and rural council clinics providing primary healthcare services, as opposed to secondary (district hospitals), tertiary (provincial), and quaternary (central) health centers, which were the settings used in other studies [32, 33, 37]. Urban primary healthcare facilities are situated in high-density suburbs and rural clinics are extensively accessible. All of these clinics typically serve as the first point of contact for individuals requiring health services. The prevalence of diabetes in PLWH in South African rural settings in 2023 was 8.1% [38, 39], and a higher prevalence of diabetes (12.1%) was reported in urban areas [38, 39].

A significant prevalence of diabetes was noted in urban clinics situated in high-density suburbs (14%) compared to 0.9 prevalence in rural clinics. This could be because two of the urban clinics from the study were donor-funded to treat and supply medication to patients with HIV comorbidities, leading to a large number of patients with comorbidities knowing their diabetes diagnoses and being on record. Another possible factor for the low prevalence of DM in rural communities could be the lower consumption of fast foods. Numerous studies have demonstrated a strong relationship between food and microbiota, demonstrating how the components of various diets may directly affect gut microbiota and may contribute to the development of various diseases, such as diabetes [40–42].

Rural Africa is likely to have a disproportionately high percentage of undiagnosed diabetes, which may be primarily caused by the inverse care rule, which is a discrepancy between patients' medical needs and the availability of healthcare. Delays in diagnosis are caused by a variety of issues, including a lack of continuity of care, delayed or improper access to care, and the existence of psychiatric and other comorbidities [43] that underestimate the true prevalence of diabetes mellitus in PLWH [44]. Prioritization in strategic planning to avoid diabetes and its complications is affected by underestimating the prevalence of diabetes and a lack of knowledge about demographic and clinical characteristics in the rural population.

Concerning the variables associated with the development of DM and HIV comorbidity, our investigation highlighted that sex and occupation exhibit a statistically significant association. Women are more likely to seek health care services than males, according to the study, just 40% of males seek medical attention, which results in male patients having a lower risk of getting diabetes than their female counterparts (AOR: 0.4; 95% CI: 0.2, 0.9). This observation accentuates the gender differences that exist in the pathogenesis of DM [45–47]. Sex hormones, which have a great impact on energy, metabolism, body composition, vascular function, and inflammatory responses among women compared with men, also help explain the differences in the risk of developing diabetes [48, 49]. A study conducted in Malawi in 2018 indicated that women were more likely to be overweight or obese than men, which is related to a higher risk of developing diabetes [50]. This finding corresponds to other research that suggests that women living with HIV face a higher risk of developing DM [32, 33, 37].

Regarding occupation, our analysis revealed that self-employed patients exhibited higher odds of developing DM than their unemployed counterparts. This could be because those working have a higher probability of moving around, thereby exercising, and can afford to buy and consume a balanced diet. Patients who drink alcohol exhibit a more than twice as much higher propensity of developing DM relative to those who do not consume alcohol (AOR: 2, *p* < 0.05; 95% CI: 0.9, 4.8). Alcohol intake leads to changes in insulin secretion by pancreatic β-cells and can also mediate insulin resistance, resulting in impaired glucose metabolism that can lead to diabetes. The investigation revealed that patients with HIV who smoke have a slightly lower likelihood of developing DM in comparison to their non-smoking counterparts (AOR: 0.02, *p* < 0.05 95% CI: 0.006, 0.1).

Engaging in physical activities was associated with a lower possibility of developing DM compared to their counterparts, and this difference was statistically significant. This finding can be plausibly explained by the fact that physical activity can enhance patients' sensitivity to insulin, which in turn improves their glucose tolerance [34]. Individuals who participate in physical activities such as sports or fitness activities are less susceptible to DM [34]. This result is consistent with prior research on the prevention and management of diabetes, which has established that physical activity can reduce the incidence of DM in both HIV-positive and HIV-negative patients [51–55].

With regard to dietary habits, HIV-positive patients who consume fruits and vegetables more often have a lower risk of developing diabetes. This can be attributed to the fact that fruits and vegetables are rich in dietary fiber, which helps regulate blood sugar levels by slowing the absorption of glucose into the bloodstream. Furthermore, fruits and vegetables are highly nutritious, as they contain vitamins, minerals, and antioxidants that are key to improving insulin sensitivity and reducing the risk of developing DM [34, 56, 57]. Yiga et al. noted that dietary habits are affected by a variety of cultural factors. One's view of one’s body has an impact on how much they eat. Weight loss is a source of stigma and an indication of HIV/AIDS; however, weight gain is related to beauty, dignity, health, richness, and excellent treatment from husbands. Other identified cultural views included high social prestige given to unhealthy fast food and eating out and low social status given to fruits, vegetables, legumes, and minimally processed cereals [58].

### Strengths and limitations

This study has some limitations that are worth to be noted. The data collection process involved gathering information from 450 patients who attended eight clinics; however, it is crucial to note that the generalizability of the study findings is limited to PLWH. The focus was on HIV-positive patients; non-HIV patients were not included in the study as references. It is crucial to highlight that information on DM status was based on patients' disclosure and medical records, and the time of diagnosis of DM was not included in the study to determine whether diabetes developed after HIV diagnosis. Consequently, this approach limits the reliability of the data, as some patients could have DM and HIV, but have not yet been diagnosed. Despite the significant contribution of this study, our results cannot be generalized because we only conducted the study at eight facilities. The strengths of the study were the inclusion of traditional risk factors for NCD development, such as tobacco and alcohol use, among others.

## Conclusion

Diabetes mellitus is increasingly prevalent among PLWH and contributes significantly to the burden of disease experienced by patients with HIV. Our research indicates a high prevalence of DM/HIV comorbidities, especially in urban communities. Adopting an active screening policy for non-communicable diseases (NCDs), particularly type 2 diabetes and hypertension, is highly recommended as a component of standard HIV care. Owing to the severe lack of resources in the majority of SSA care settings, targeted screening based on factors such as age and sex may be suggested.

The rapid scaling of treatment delivery in the face of severe human capacity constraints can be made possible by task shifting of HIV care in PHC clinics from doctors to nurses and other less-skilled cadres. Despite these achievements, frontline professional training is recommended to keep up with the changing epidemic and its new issues. The adoption of an integrated health model that comprehensively addresses all chronic diseases in patients at the primary care level may help improve the quality of health services provided to PLWH with comorbidities. Early detection of NCD by opportunistic screening of PLWH will facilitate early management and potentially reduce or delay the onset of cardiovascular disease.

### Supplementary Information

**Additional file 1:** **Appendix 3.** English Questionnaire for Objective 2.

## Data Availability

Data and materials are available.

## Abbreviations

HAART: Highly active antiretroviral treatment
HIV: Human immunodeficiency virus
CD: Chronic disease
CI: Confidence intervals
DM: Diabetes mellitus
HIC: High-income countries
HIV: Human immunodeficiency virus
LMIC: Low- and middle-income countries
NCDs: Non-communicable diseases
NGOs: Non-governmental organizations
PCNs: Primary Care Nurses
PHC: Primary healthcare
PLWH: People living with HIV
SPSS: Statistical Package for Social Science
SSA: Sub-Saharan Africa
T2DM: Type 2 Diabetes Mellitus
VHWs: Village Health Workers
WHO: World Health Organization
