# Supplementary material for: Diabetes mellitus and associated factors among HIV-positive patients at primary health care facilities in Harare, Zimbabwe: a descriptive cross-sectional study
Source: BMC Prim Care. 2024 Jan 15;25:28. doi: 10.1186/s12875-024-02261-3 (PMC10789024; doi:10.1186/s12875-024-02261-3)
Supplement: Supplementary file 1 — Additional file 1: Appendix 3. English Questionnaire for Objective 2. [file 12875_2024_2261_MOESM1_ESM.docx]

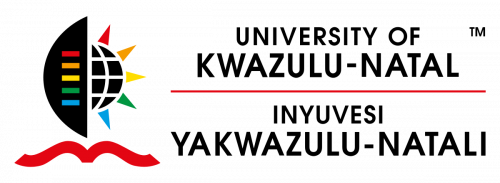


## Appendix 3: English Questionnaire for Objective 2

## Title: Prevalence of diabetes mellitus and associated factors among HIV-positive patients at primary health care facilities in Harare, Zimbabwe

## *Survey of Chronic Disease Services*

Please answer the following questions by placing a tick for the correct and applicable answer.

**Participants’ questionnaire**:

1. Centre …………………
2. Participant identification number ………
3. Phone number (*for contact if need be*) …………
4. Date of completion of questionnaire …………
5. Consent has been read and accepted Yes [ ] No [ ]

**SECTION A. DEMOGRAPHIC INFORMATION**

1. Gender 1. Male [ ] 2. Female [ ] 3. Other [ ]
2. Age 1. [<35] [ ] 2. [>35] [ ]
3. What is the highest level of education that you have obtained
4. primary school [ ]
5. Secondary school [ ]
6. Higher education [ ]
7. No formal education [ ]
8. Marital status: 1. Married [ ] 2. Single/Not married [ ]  3. Other………………
9. What is your ethnic group: 1. Black [ ] 2. Indian [ ] 3. White [ ] 4. Other []………………
10. Religion: 1. Christianity [ ] 2. Muslim [ ] 3. Hindu [ ] 4. Other [ ] ……..
11. Employment status: 1. unemployed [ ], 2. self-employed [ ], 3. formally employed [ ]

**SECTION B. Behavioural Measurements**

**Smoking**

1. Do you CURRENTLY smoke any tobacco products? 1. YES [ ] 2. NO [ ]

**Alcohol consumption**

1. Have you ever consumed an alcoholic drink? 1. YES [ ] 2. NO [ ]

**Diet**

1. How many servings of fruit and vegetables per day?

Not daily [ ]

Once a day [ ]

Twice per day [ ]

More often [ ]

***Balanced diet****: a diet consisting of a variety of different foods that provide adequate amounts of nutrients necessary for good health. For example, fruits, vegetables, whole grains eg sadza, rice, lean protein eg chicken and meat with no fat, nuts and legumes. These foods are to be consumed together per one sitting in reasonable quantities.*

15.1. Do you have a prescribed diet? 1. Yes [ ] 2. No [ ]

If Yes, what is it? ……………………………………

**Physical Activity**

1. How many days do you exercise (thus any bodily activity that enhances or maintains physical fitness and overall health and wellness for example running, walking, Zumba, weightlifting etc.) in a typical week?

1 day per week [ ]

2 to 3 days per week [ ]

4 to 5 days per week [ ]

6 to 7 days per week [ ]

Duration and intensity of exercise

30 min [ ]

45 min [ ]

60 minutes [ ]

1. Does your work include activities that cause a significant increase in breathing or heart rate *[ e.g. carrying or lifting* *heavy weights, digging or construction work]* for at least 10 minutes without a break? 1. Yes [ ] 2. No [ ]

**SECTION C. Medical history**

**Disease and Treatment History**

1. Have you ever been diagnosed with any of the following diseases?
2. Hypertension (BP) 1. Yes [ ] 2. No [ ]
3. Stroke 1. Yes [ ] 2. No [ ]
4. Cancer 1. Yes [ ] 2. No [ ]
5. Raised blood cholesterol 1. Yes [ ] 2. No [ ]
6. (Heart condition) 1. Yes [ ] 2. No [ ]
7. Other ……………………………………………………….
8. Any family history of diabetes? 1. Yes [ ] 2. No [ ]
9. Have you been diagnosed with diabetes? 1. Yes [ ] 2. No [ ]

IF YES,

20.1. What medication are you using to manage diabetes?.......................

20.2. Do you have a prescribed diet for this condition?

1. Yes [ ] 2. No [ ]

20.2.1. If YES what is the diet? …………………………………………………………………………

20.3. Have you received any other health education on diabetes, for example, foot care, or home blood sugar monitoring?

1. Yes [ ]

2. No [ ]

20.3.1. IF YES, describe ……………………………………………………………………………………………………………………………………………………………………………………

20.4. Have you been referred to any other health professional?

1. Yes [ ]

2. No [ ]

20.4.1. If Yes then who? ……………………………………………………………………………………………………………………………………………………………………………………

**History of HIV**

1. How long have you been diagnosed? Years …….. Months …… weeks ……
2. Have you been initiated on ARVs? 1. Yes [ ] 2. No [ ]
3. If YES what HAART medication are you taking? ……………………………
4. How long do you usually wait in line to get medical care service at the clinic? hours [ ] Minutes [ ]

***Waiting times differ depending on the service one is receiving from the health centre.***

**For patients with comorbidities (Yes for question 20)**

1. 1. Did you manage to get help for both conditions at the same clinic? 1. Yes [ ] 2. No [ ]

25.2. Was it from the same department? 1. Yes [ ] 2. NO [ ]

1. Are the conditions, HIV/AIDS and T2DM treated separately? 1. Yes [ ] 2. No [ ]
2. Did you manage to get health education on both conditions? 1. Yes [ ] 2. No [ ]
3. Was every procedure explained to you so that you can be part of the process? 1. Yes [ ] 2. No [ ]
4. Were you referred to other departments to receive other medical services e.g., to the laboratory for blood collection? 1. Yes [ ] 2. No [ ]

If yes, which department were you referred to?............................................

1. Was blood for tests collected on separate occasions? 1. Yes [ ] 2. No [ ]
2. Did you manage to ask questions about your conditions? 1. Yes [ ] 2. No [ ]
3. What do you think about the current way of management of patients like you?.................................................................................................

**SECTION D. Physical and Biochemical Measurements**

1. Height ……………………
2. Weight ……………………..
3. **For women**: Are you pregnant 1. Yes [ ] 2. No [ ]
4. Blood pressure reading …………………………
5. Heart rate ……………………..
6. for the last 12 hours? Yes [ ] No [ ]
7. Blood glucose level……………………
8. Recent HbA1C results …………………………………
